# Disparity Expression of Notch1 in Benign and Malignant Colorectal Diseases

**DOI:** 10.1371/journal.pone.0081005

**Published:** 2013-12-03

**Authors:** Rui Huang, Qingchao Tang, Qi You, Zheng Liu, Guiyu Wang, Yinggang Chen, Yuwen Sun, Shan Muhammad, Xishan Wang

**Affiliations:** 1 Department of Colorectal Surgery, The Second Affiliated Hospital of Harbin Medical University, Harbin, China; 2 Department of Pathology, The Second Affiliated Hospital of Harbin Medical University, Harbin, China; 3 Colorectal Cancer Institute of Harbin Medical University, Harbin, China; Shanghai Jiao Tong University School of Medicine, China

## Abstract

**Background and Objectives:**

Although there was growing evidence supporting the hypothesis that Notch1 was one of the few candidate genes linked with colorectal cancer (CRC) susceptibility, the precise level of Notch1 protein expression in benign and malignant colorectal diseases was still unknown. Our study has investigated the Notch1 expression in benign and malignant colorectal diseases as well as to investigate the role and clinicopathological significance of aberrant expression of Notch1 in CRC.

**Methods:**

The protein expression of Notch1 was examined by immunohistochemistry in 901 clinical specimens with colorectal diseases, including 220 patients with ulcerative colitis, 232 patients with colorectal adenoma and 449 patients with colorectal cancer. Associations between the expression of Notch1 and various clinicopathological features, as well as survival status, were studied.

**Results:**

Cytoplasmic Notch1 was expressed in 7.7% of patients with ulcerative colitis, 14.7% of patients with colorectal adenoma and 58.0% of patients with colorectal cancer, respectively. Colorectal cancer patients with high expression levels of Notch1 showed lower overall survival (OS) and disease-free survival (DFS) rates than those patients with low Notch1 expression.

**Conclusions:**

Expression level of Notch1 was gradually increased from precancerous lesions to cancer. It might play as an oncogene in the CRC development, and might be potentially used as a biomarker for prognosis of CRCs.

## Introduction

Colorectal cancer (CRC) is among the most frequent types of malignancy. In terms of incidence, it is the third most common cancer worldwide [1]. Because colorectal cancer in its early stages is difficult to diagnose, the prognosis is poor. Therefore, early diagnosis and prediction of prognosis of colorectal cancer has become particularly important. Ulcerative colitis and colorectal adenoma are benign bowel diseases, but there is the possibility of canceration [2]–[4]. The occurrence and development of colorectal cancer are closely related to precancerous lesions [5]. Thus, studying the relationship between precancerous lesions and tumors plays an important role in the prevention of colorectal cancer.

Notch signaling is an important molecular pathway and plays a critical role in maintaining the progenitor/stem cell population, as well as keeping a balance between cell proliferation, differentiation and apoptosis. Therefore, Notch signaling may be involved in carcinogenesis. In humans, the Notch signaling pathway consists of five Notch ligands (Delta-like 1, 3, 4 and Jagged 1, 2), four Notch receptors (Notch 1–4) and several downstream target genes such as p21, Hes-1and Deltex [6], [7]. Notch1 represents the first link of the Notch cascade to human cancer.

Recent years Notch1 is identified as an oncogene responsible for acute T cell lymphoblastic leukemia (T-ALL) [8], and its oncogenic effects are also found in glioma, primary melanoma and pancreatic cancer [9]–[11]. It must be noted that Notch signaling does not always function as an oncogenic factor. Conversely, Notch1 may act as a tumor suppressor in mouse skin [12]. Klinakis A [13] show that silencing Notch activity leads to the development of myeloid leukaemia, implying a novel tumor-suppressor function for the Notch pathway in haematopoiesis. Similarly, the precise role of Notch signaling in CRCs is controversial [14], [15]. Specifically, the function of Notch1 expression is unclear in colorectal precancerous lesions. Therefore, we investigate the notch1 expression in benign and malignant colorectal diseases and try to clarify that this signaling molecule plays an important oncogene role in the development of CRCs.

## Materials and Methods

### Ethic statement

All protocols were reviewed and approved by the Ethical Committee of Harbin Medical University in Harbin, China. Participants provided their written informed consent to participate in this study.

### Patient selection and specimens

Nine hundred and one patients with colorectal disease were included in this study, from January 2005 till January 2007 at the Second Affiliated Hospital of Harbin Medical University, China. The immunohistochemical analysis was performed from 220 ulcerative colitis patients, 232 colorectal adenoma patients, and 449 colorectal cancer patients. The sample consisted of 452 patients who underwent routine screening colonoscopy and were diagnosed with ulcerative colitis and colorectal adenoma, excluding patients with current or previous malignant neoplasms, intestinal polyposis, prior radiotherapy. In the colorectal cancer group 12 of 449 patients have a history of the benign colon lesions, 9 patients with ulcerative colitis, 3 patients with colorectal adenoma. Others did not undergo precancerous screening procedures. None of the colorectal cancer patients recruited in this study received chemotherapy or radiotherapy before surgery. After reviewing the pathological information and medical records, available cases for IHC with paraffin blocks were collected. The pathologic reviews were double blinded and performed by two pathologists who were experienced in colorectal diseases pathology. Outcomes were determined from the date of colonoscopy or surgery until January 2012, which resulted in a follow-up period of from 60 to 84 months. Fifty five cases (lost to follow-up or died from causes other than CRC) were censored during survival analysis. Follow-up data were retrieved from the computerized archive of health care services of our institutions and confirmed by death certificates and direct interviews with their relatives. All colorectal cancer patients were adenocarcinomas. Clinicopathological parameters such as age, sex, LNM (lymph node metastasis), infiltrate depth, differentiation, tumor location, TNM pathologic stage according to American Joint Committee on Cancer classification were evaluated by reviewing medical and pathological records.

### Immunohistochemistry and Interpretation

IHC was performed on 4-µm sections that were prepared from paraffin-embedded tissue arrays. Following standard procedures, all sections were deparaffinized with xylene, dehydrated through graded alcohol before endogenous peroxidase activity was blocked with 3% H_2_O_2_ in methanol for 30 min. Then, the sections were pretreated with Tris/ethylene diamine tetraacetic acid (EDTA) buffer with a pH of 9.0 at 100°C for 10 min and incubated overnight at 4°C in primary anti- Notch1 monoclonal antibody (1∶100, Abcam, Cambridge, UK). Next, the sections were treated with biotinylated secondary antibody for 30 min, followed by further incubated in streptavidin-peroxidase complex for 30 minutes, and visualized with 0.05% 3,3-diaminobenzidine tetrahydrochloride. Finally the sections were counterstained with hematoxylin. The primary antibody substituted with PBS was used as negative control. The positive control was brain neoplasm with positive expression of Notch1 [16].

Semi quantitative expression levels were based on the Notch1 staining intensity and distribution. Cytoplasmic staining intensity was graded as 0 (no staining), 1 (weak staining = light yellow), 2 (moderate staining = yellow brown), and 3 (strong staining = brown) (Figure S1). The percentage of the extent of reactivity was scored as follows: 0 (no positive cells), 1 (less than 10% positive cells), 2 (10–50% positive cells), and 3 (more than 50% positive cells) [17]. Then, the cytoplasmic expression score was obtained by multiplying the intensity and reactivity extension values. Score<1 was classified as negative. Scores<4 were classified as low expression, and the others were classified as high expression.

### Statistical analysis

All data were expressed as mean ± standard error of the mean. Associations between Notch1 expression and categorical variables were assessed by the Chi-square test. Survival curve was estimated using the Kaplan–Meier method. Statistical analyses were performed by using SPSS for Windows, Version 10.0 software package. *P*<0.05 was considered as significant.

## Results

### Patients' characteristics

Of the 971 patients approached, 956 patients agreed to participate in the study (98%). Of these, a total of 901 patients between 38 and 90 years old were found to be suitable for inclusion: 220 patients with ulcerative colitis, 232 patients with colorectal adenoma, and 449 patients with CRC. Among the patients with UC, 52 had pancolitis, 78 had left-side disease, and 90 had proctitis. Of these 220 patients, 187 (85%) were admitted because of an aggravation of symptoms associated with colitis. In the colorectal adenoma group, a total number of 272 colorectal adenomas samples came from 232 patients, and 40 of 232 patients had two adenomas simultaneously. The polyps were distributed as follows: 52 (19%) were located in the rectum and 220 (81%) in the colon. The size of the polyps ranged from 5 mm to 24 mm (average: 6.4±4.8 mm). With the exception of one case of tubulo-villous adenoma, all other cases were histologically classified as tubular adenoma. All of these patients were followed up more than 5 years after samples were collected (median follow up 62 months), during which time no dysplasia or cancer was detected. A total number of 462 CRCs samples came from 449 patients who underwent surgery. In the colorectal cancer group 13 of 449 patients had two colorectal cancer lesions simultaneously. (Table 1 and 2)

**Table 1:** Summary of the clinicopathologic features of ulcerative colitis, adenoma and carcinoma patients.

	No. of ulcerative colitis patients	No. of colorectal adenoma patients	No. of colorectal cancer patients
Number of patients	220	232	449
Male/Female	115/105	117/115	248/201
Age			
Median	46.3	48.2	59.4
Range	30–72	31–78	30–81
Number of lesions	–	272	462
Location			
colon	–	220	256
Rectum	–	52	206
Extent			
Proctitis	52	–	–
Left sided	78	–	–
Subtotal/total	90	–	–

**Table 2:** Correlation between Notch1 expression and clinicopathological features in patients with colorectal cancer.

		Notch1 Expression		
Characteristic	Number	Negative	Positive	*P*
Age				0.311
≤60	233	95 (40.8)	138 (59.2)	
>60	216	94 (43.5)	122 (56.5)	
Sex				0.163
male	248	110 (44.4)	138 (55.6)	
female	201	79 (39.3)	122 (60.7)	
LNM				0.067
negative	256	116 (45.3)	140 (54.7)	
positive	193	73 (37.8)	120 (62.2)	
TNM stage				0.024
I	84	35 (41.7)	49 (58.3)	
II	165	83 (50.3)	82 (49.7)	
III	145	55 (37.9)	90 (62.1)	
IV	55	16 (29.1)	39 (70.9)	
Infiltrate depth				0.018
T1+T2	234	110 (47.0)	124 (53.0)	
T3+T4	215	79 (36.7)	136 (63.3)	
Differentiation				0.007
well	159	81 (50.9)	78 (49.1)	
moderately	194	78 (40.2)	116 (59.8)	
poorly	96	30 (31.2)	66 (68.8)	
Tumor location				0.408
rectum	206	85 (41.3)	121 (58.7)	
colon	243	104(42.8)	139(57.2)	

LNM, Lymph node metastasis; TNM, tumor node metastasis.

### Expression of Notch1 in colorectal cancer compared with that in benign tissues

**Figure 1:**
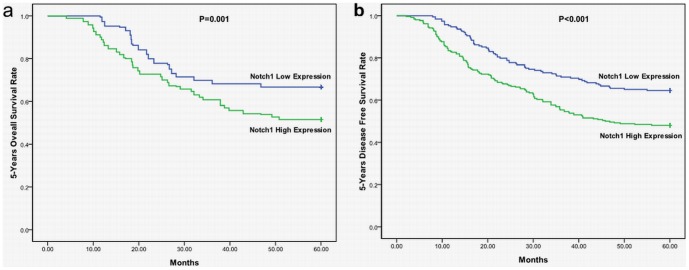
Immunohistochemical staining of Notch1 in colorectal tissues (400×). a: normal adjacent colorectal specimen. b: ulcerative colitis Notch1-low-expression specimen. c: adenoma Notch1-low-expression specimen. d: Strongly immunoreactive cytoplasmic in a tumor with high Notch1 expression.

Expression of Notch1 was examined in the cytoplasm of benign tissues (normal adjacent, ulcerative colitis, adenoma tissue) (Fig. 1 a, b and c) and cancer tissues (Fig. 1 d). Overall, colorectal cancer tissues exhibited dramatically higher levels of Notch1 protein expression compared with benign tissues (*P*<0.001) (Table 3). High expression of Notch1 (Fig. 1 d) was found in 268 of 462 (58.0%) colorectal cancer tissues. Of the 272 colorectal adenoma specimens, we found high expression in 40 (14.7%) samples. High expression was also detected in ulcerative colitis (7.7%) tissues. All clinical features of the benign lesions with high Notch1 expression were indicated in Table S1. There were significantly more cases (110 and 135) with low expression of Notch1 in ulcerative colitis (Fig. 1 b) and adenoma tissue (Fig. 1 c). In normal adjacent controls, we had not found high cytoplasmic notch1 expression. Only 2 of 449 normal adjacent tissues were detected with a low level of Notch 1, remains of them were negative in Notch 1 expression (Fig. 1 a). Synchronous lesions had the same levels of Notch1 protein expression.

**Table 3:** Notch1 expression level in three groups.

			Notch1 Expression		
Type	Number	Negative	Low	High	*P*
Ulcerative colitis	220	93(42.3)	110 (50.0)	17 (7.7)	0.001
Colorectal adenoma	272	97(35.7)	135(49.6)	40(14.7)	
Cancer	462	23(5.0)	171(37.0)	268(58.0)	

### Relationship between expression of Notch1 and clinicopathological features

We analyzed the associations between levels of Notch1 expression and a series of clinicopathological characteristic, including age, sex, LNM, infiltrate depth, differentiation, tumor location, TNM pathologic stage, in colorectal cancer patients (Table 2). A total of 136 of 215 patients whose tumor infiltrate depth was T3+T4 had significantly higher incidences of high Notch1 expression than those patients with a tumor infiltrate depth T1+T2 (53.0%) (*P* = 0.018). We also detected high Notch1expression in 49 of 84 (58.3%) stage I patients, 82 of 165 (49.6%) stage II patients, 90 of 145 (62.1%) stage III patients and 39 of the 55 (70.9%) stage IV patients. These data demonstrated that frequency and intensity of Notch1 expression were greater in late-stage than in early-stage colorectal cancer (*P* = 0.024). The expressions of Notch1 were statistically correlated with tumor differentiation status. Notch1 expression was up-regulated in tumors with poor differentiation (*P* = 0.007). We found that there were no significant correlation between Notch1 cytoplasmic levels and clinicopathological levels, including the patient's age (*P* = 0.311), sex (*P* = 0.163), LMN (*P* = 0.067), and tumor location (*P* = 0.408).

### Survival analysis of Notch1 expression in patients with colorectal cancer

**Figure 2:**
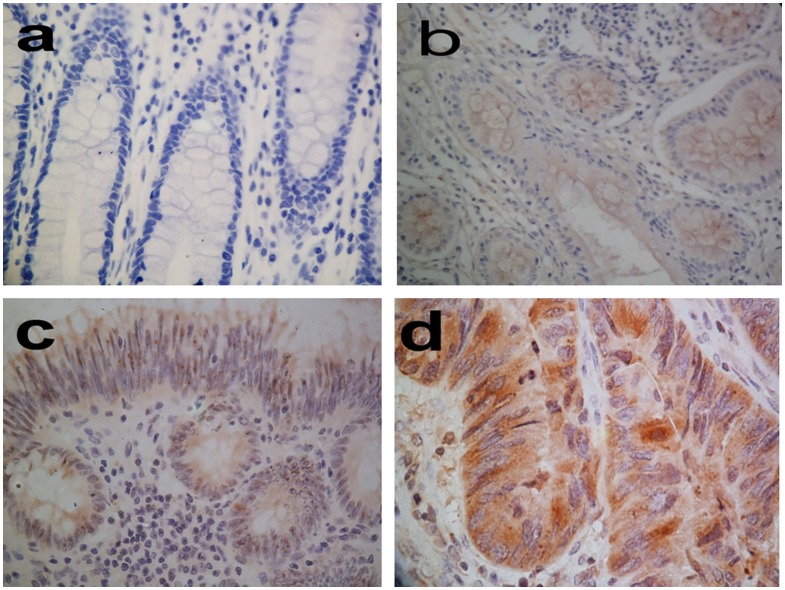
Kaplan–Meier curves illustrating correlation of Notch1 expression with survival (a) overall survival (OS) (log-rank test, *P* = 0.001) and (b) disease-free survival (DFS) (log-rank test, *P*<0.001).

The Kaplan–Meier 5-year survival curves, stratified for Notch1 expression, were shown in Figure 2. Among the 449 study patients, the scores of cytoplasmic expression showed significant effects on OS (*P* = 0.001) and DFS (*P*<0.001). These data indicated that high Notch1 expression was associated with worse OS and worse DFS.

Multivariate survival analysis was performed by testing adverse factors identified in univariate analysis in the Cox model. Only Notch1 expression (*P* = 0.003, Hazard Ratio 1.577, 95%CI 1.164–2.139) retained prognostic significance for 5y-OS. Notch1 expression (*P* = 0.001, Hazard Ratio 1.676, 95%CI 1.248–2.251) had prognostic significance for 5y-DFS.

## Discussion

Notch signaling had different functions among different tumors. On the one hand, Notch signaling was often considered a model proto-oncogene because of its role as the main trigger of tumors, including leukemia and solid tumors. On the other hand, growing evidence showed that Notch signaling could also have a potent tumor suppressor function in many tumors. Nicolas [12] suggested that Notch acted as a tumor suppressor in the skin through suppression of the Wnt and Sonic–hedgehog pathways. Recent study suggested that silencing Notch activity led to the development of myeloid leukaemia, implying a novel tumor suppressor function for Notch pathway in haematopoiesis [18].

Sporadic CRC might initiate from colonic precancerous lesions such as inflammatory bowel diseases and adenomatous polyps. However, the role of Notch signaling in these precancerous colorectal lesions had been rarely studied. Rodilla [19] found that Jagged1 messenger RNA was expressed at a significant higher level in adenomas of patients with familial adenomatous polyposis. Overexpression of Notch1 was observed in the dextran sodium sulfate-induced ulcerative colitis in mice [20]. We speculated that activated Notch signaling might be related to the increased susceptibility of colorectal cancer development in some precancerous conditions.

Because of the complexity of the Notch signaling pathway, we tried to elucidate its role in colorectal diseases. Though Notch1 had been shown to be activated in colorectal cancer [21], its expression in benign colorectal patients was still uncertain. We first investigated the cytoplasmic expression of Notch1 in human colorectal adenoma tissues and in ulcerative colitis tissues at the level of protein by using IHC method. We found that Notch1 was expressed at low level in these tissues. But we also observed a few high expression of Notch1 in the benign diseases. It was clear that patients with long-term ulcerative colitis had an increased risk of colorectal cancer, and the risk rose with approximately 0.5–1.0%/yr [22]. Colorectal adenoma subsequent progression to carcinoma occurred in about 5% of the cases [23]. In addition, recent research indicated that active Notch signaling might be the major driving force behind the proliferative potential of adenomas and adenocarcinomas of the intestine [24]. All of these patients were followed up for more than 5 years, during which time no cancer was detected. Then, we considered two reasons that Notch1 high expressing benign lesions did not progress to CRCs during five years of follow-up. First, five years of follow-up might be too short and it was not enough to make benign lesions progress to CRCs even if these patients had more risk of tumorigenesis. Therefore, we still need to follow up these patients, especially those patients with high notch1 expression level. Second, a larger sample size might be necessary to better characterize the question. We then observed that Notch1 was overexpressed in colorectal cancer tissues. This might reflect cytoplasmic signal activation of Notch1 for regulating the growth of colorectal adenocarcinomas. Our results were identical with the reported by Kim HA [14], but our results conflicted with the reported by Zhang Y [15]. Activation mutations of Notch1 occurred relatively frequently in T-cell leukemia [25]. It might be expected that such mutations also occurred in colorectal cancer. Although the mechanism was unclear, a number of recent researches had shown that Notch1 activation could induce the expression Hes3 and sonic hedgehog (Shh) by activating cytoplasmic serine/threonine kinase Akt, signal transducer and activator of transcription 3 (ATAT3), and mammalian target of rapamycin (mTOR), promoting the survival of neural stem cells and inducing brain cancer [18]. The CRC development and progression could be step by step genetic modification resulted in benign lesions to carcinoma [26]. Here we provided further evidence supporting these views and implicated that altered Notch1 expression in human colorectal tissues increased the propensity for tumor formation.

Finally, we analyzed Notch1 expression in 449 patients with CRC. A statistically important relevance was found in our research regarding larger infiltrate depth with higher Notch1 expression. Furthermore, our results revealed that, besides infiltrate depth, clinicopathological staging was also associated with Notch1 expression. Other factors, such as age, gender, LMN, and tumor location, were not associated with Notch expression. Moreover, high Notch1 expression was related to poor OS and DFS. Our observations showed that Notch1 might become a valuable predictor for prognosis and survival among colorectal cancer patients. These results were in support of a potential role of Notch signaling in CRC formation, and Notch1 was probably an important regulator for CRC formation. More studies were needed to clarify the regulatory role and molecular mechanisms of Notch1 in CRC.

In conclusion, the results of this study showed that the expression level of Notch1 may be gradually increased from precancerous lesions to cancer. In addition, our data demonstrated a significant relationship between high expression of Notch1 protein and poor prognosis in patients with CRC. Conceivably, Notch1 may act as an oncogene during the CRC development, and might be used as a potential biomarker for prognosis and therapy of CRCs.

## Supporting Information

Figure S1 **Protein immunohistochemistry.** a: negative. b: low expression. c: high expression.
(TIF)

Table S1 **Clinical features of the benign lesions with high Notch1 expression in this study.**
(DOCX)
