# Supplementary figures and images for: Disparity Expression of Notch1 in Benign and Malignant Colorectal Diseases
Source: PLoS One. 2013 Dec 3;8(12):e81005. doi: 10.1371/journal.pone.0081005 (PMC3849093; doi:10.1371/journal.pone.0081005)

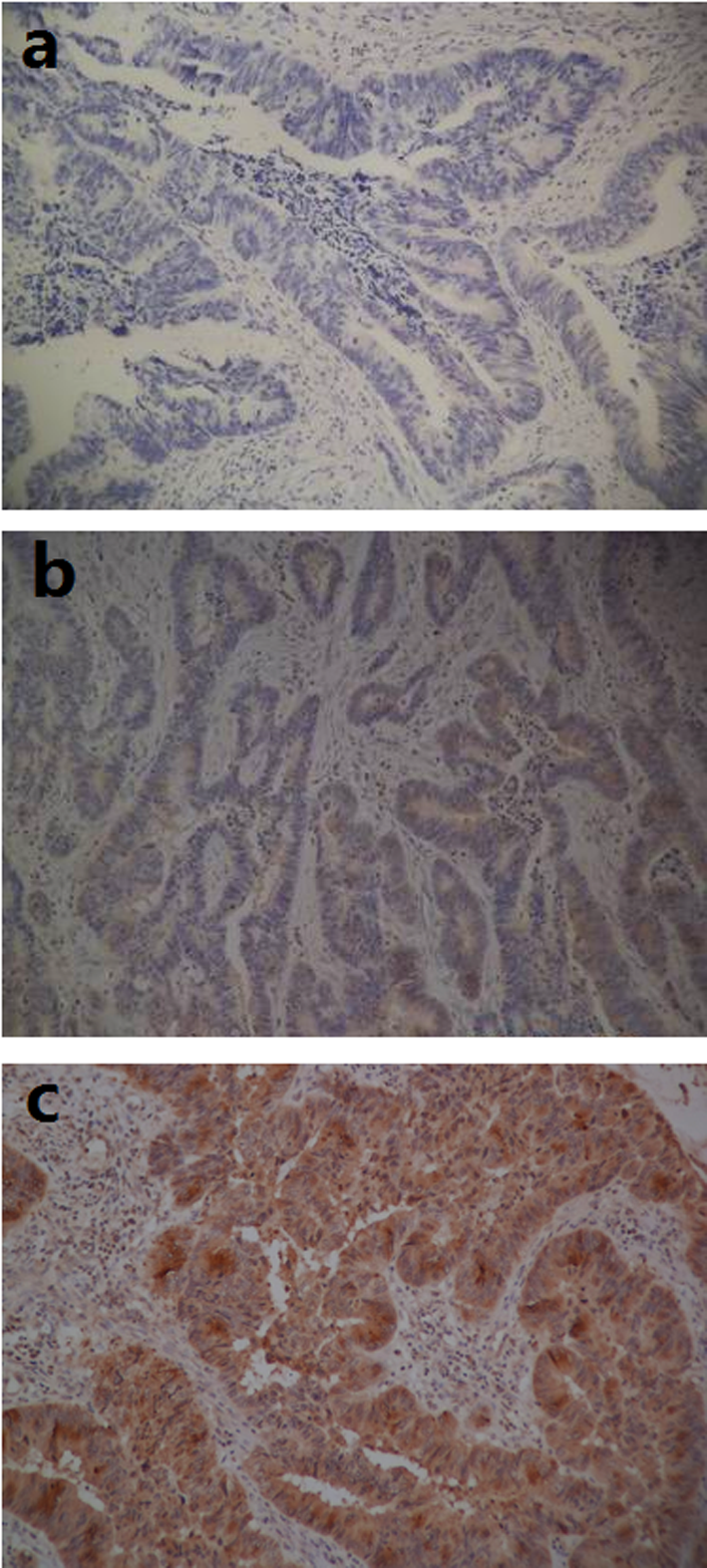

Supplement: Figure S1 — Protein immunohistochemistry. a: negative. b: low expression. c: high expression. (TIF) [file pone.0081005.s001.tif]
