# Supplementary material for: Disparity Expression of Notch1 in Benign and Malignant Colorectal Diseases
Source: PLoS One. 2013 Dec 3;8(12):e81005. doi: 10.1371/journal.pone.0081005 (PMC3849093; doi:10.1371/journal.pone.0081005)
Supplement: Table S1 — Clinical features of the benign lesions with high Notch1 expression in this study. (DOCX) [file pone.0081005.s002.docx]

Table S1 Clinical features of the benign lesions with high Notch1 expression in this study

|  | Ulcerative colitis patients | Colorectal  adenoma patients |
| --- | --- | --- |
| Number of patients | 17 | 40 |
| Male/Female | 8/9 | 23/17 |
| Age |  |  |
| Median | 51.3 | 53.6 |
| Range | 38－71 | 33－75 |
| average size (mm) | － | 11.2 |
| Location |  |  |
| colon | － | 31 |
| Rectum | － | 9 |
| Extent |  |  |
| Proctitis | 1 | － |
| Left sided | 5 | － |
| Subtotal/total | 11 | － |
